# Resveratrol attenuates staphylococcal enterotoxin B-activated immune cell metabolism *via* upregulation of miR-100 and suppression of mTOR signaling pathway

**DOI:** 10.3389/fphar.2023.1106733

**Published:** 2023-02-24

**Authors:** Hasan Alghetaa, Amira Mohammed, Narendra Singh, Kiesha Wilson, Goushuai Cai, Nagireddy Putluri, Mitzi Nagarkatti, Prakash Nagarkatti

**Affiliations:** ^1^ Department of Physiology, Biochemistry and Pharmacology, College of Veterinary Medicine, University of Baghdad, Baghdad, Iraq; ^2^ Department of Pathology, Microbiology and Immunology, School of Medicine, University of South Carolina, Columbia, SC, United States; ^3^ Department of Environmental Health Sciences, Arnold School of Public Health, University of South Carolina, Columbia, SC, United States; ^4^ Dan L. Duncan Cancer Center, Advanced Technology Core, Alkek Center for Molecular Discovery, Baylor College of Medicine, Houston, TX, United States

**Keywords:** MiR-100, ARDS (acute respiratory disease syndrome), T-cell metabolism, mTOR, staphylococcal enterotoxin B (SEB), resveratrol, metabolome, ScRNA

## Abstract

Acute Respiratory Distress Syndrome (ARDS) is triggered by a variety of insults, such as bacterial and viral infections, including SARS-CoV-2, leading to high mortality. In the murine model of ARDS induced by Staphylococcal enterotoxin-B (SEB), our previous studies showed that while SEB triggered 100% mortality, treatment with Resveratrol (RES) completely prevented such mortality by attenuating inflammation in the lungs. In the current study, we investigated the metabolic profile of SEB-activated immune cells in the lungs following treatment with RES. RES-treated mice had higher expression of miR-100 in the lung mononuclear cells (MNCs), which targeted mTOR, leading to its decreased expression. Also, Single-cell RNA-seq (scRNA seq) unveiled the decreased expression of mTOR in a variety of immune cells in the lungs. There was also an increase in glycolytic and mitochondrial respiration in the cells from SEB + VEH group in comparison with SEB + RES group. Together these data suggested that RES alters the metabolic reprogramming of SEB-activated immune cells, through suppression of mTOR activation and its down- and upstream effects on energy metabolism. Also, miR-100 could serve as novel potential therapeutic molecule in the amelioration of ARDS.

## Introduction

Acute respiratory distress syndrome (ARDS) is a serious clinical disorder triggered by many etiological agents such as bacterial, fungal and viral infections as well as certain chemical toxicants (de Prost et al., 2017). ARDS is characterized by fluid leaking into the lungs, difficulties in breathing, low blood oxygenation rate and is defined as a life-threatening lung injury. Additionally, ARDS is associated with hyperactivation of the immune response in the lungs, and systemically. This often leads to cytokine secretion, leading to the development of pulmonary edema, alveolar damage, and respiratory as well as multiorgan failure (Nagarkatti et al., 2020). ARDS is extremely challenging to treat because of which almost 40% of people with ARDS die (Dembinski and Mielck, 2018). The pandemic caused by SARS-CoV-2 virus (COVID-19) is also well characterized to trigger ARDS leading to high levels of mortality (Perico et al., 2021). Currently, there are no pharmacological agents approved by the Food and Drug Administration to specifically treat ARDS.

Staphylococcal enterotoxin-B (SEB) is one of many toxins secreted by *Staphylococcus aureus* bacterium and can cause food poisoning outbreak when ingested with contaminated food leading to vomiting, diarrhea and different gastrointestinal symptoms (Ercoli et al., 2017; Guidi et al., 2018). SEB can also cause ARDS when aerosolized because of which it is classified as select agent of biological warfare by CDC (Pohanka, 2019). SEB activates a subfamily of T cells that express Vβ7 and Vβ8 on their T cell receptors thereby activating a significant proportion of T cells to produce cytokines (Sultan et al., 2021). Thus, inhalation of SEB leads to pro-inflammatory cytokine storm systemically (Fries and Varshney, 2013; Popugailo et al., 2019; Mohammed et al., 2020c), which in turn triggers massive proliferation and recruitment of effector T cells, macrophages, and neutrophils from the periphery into the lungs leading lung injury (Savransky et al., 2003; Muralimohan et al., 2008; Rao et al., 2015b; Mohammed et al., 2020c; Ferreira-Duarte et al., 2020). This could also occur due to microRNA dysregulation in immune cells that will lead to hyperactivity of immune cells, especially the T effector cells (Alghetaa et al., 2018; Mohammed et al., 2020a; Mohammed et al., 2020c).

microRNA (miRNA, miR) are small non-coding RNA molecules (∼22 nucleotides) that regulate mRNA expression involved in different biological cellular functions such as cell death, cell proliferation and cell metabolism (Ambros, 2004). Recently, several studies have demonstrated aberrant levels of miRNAs associated with ARDS, indicating their complex role in regulating the etiopathology of ARDS (Zhu et al., 2017; Alghetaa et al., 2018; Ferruelo et al., 2018; Wu et al., 2019; Mohammed et al., 2020a). Previous studies from our laboratory found that miRNAs dysregulation mediated by SEB involved multiple pathways that exacerbated the inflammation in the lungs including suppression of T cell apoptosis (Rao et al., 2015a; Elliott et al., 2016; Mohammed et al., 2020a), suppression of TGF-β and T reg signaling (Rao et al., 2015b; Alghetaa et al., 2018), inhibition of SOCS1 (Rao et al., 2014) and increase NF-_K_B signaling (Rieder et al., 2012). Interestingly, we found that natural products such as cannabinoids and resveratrol reversed the dysregulation caused by SEB, attenuated the ARDS and protected the lungs (Alghetaa et al., 2018; Mohammed et al., 2020a; Mohammed et al., 2020b; Alghetaa et al., 2021).

Previous studies showed that Resveratrol (RES; trans-3,5,4′-trihydroxystilbene), a phytoalexin, was found to prevent SEB-induced ARDS and mortality in mice (Alghetaa et al., 2018; Alghetaa et al., 2021). RES is present in various foods such as grapes, red wine, pistachios, peanuts, blueberries and dark chocolate (Tian and Liu, 2020). Several studies have suggested that resveratrol may be effective in the prevention and treatment of delayed type of hypersensitivity, injured testes, aging and age-related diseases, colitis and colorectal cancers (Alrafas et al., 2019; Khayoon and Al-Rekabi, 2020; Abdulla et al., 2021; Zhou et al., 2021; Abdulla and Al-Okaily, 2022; Al-Salman et al., 2022). While this effect may be mediated through several pathways, one of them may involve mechanistic/mammalian target of rapamycin (mTOR). mTOR is involved in the regulation of aging as well as other functions such as inflammation (Papadopoli et al., 2019). Interestingly, resveratrol has been shown to inhibit mTOR signaling (Liu et al., 2010).

In the current study, we tested the central hypothesis that resveratrol may suppress SEB-mediated hyperactivation of the immune cells through the downregulation of mTOR signaling pathway. We found that while SEB upregulated the expression of mTOR in T cells, resveratrol reversed this action. We also identified miR-100-5p as the key regulator of the mTOR expression induced by resveratrol.

## Materials and methods

### Experimental animals

Female C3H/HeJ mice 6-8-week-old were purchased from The Jackson Laboratory. All mice were housed in specific pathogen–free conditions at the Association for Assessment and Accreditation of Laboratory Animal Care Internationally Accredited University of South Carolina, School of Medicine, Animal Resource Facility and were kept under 12 light/12 dark cycles at a temperature of ∼18°C–23°C and 40%–60% humidity. Food and water were available *ad libitum*. All experiments performed using mice in this study were approved by the Institutional Animal Care and Use Committee, University of South Carolina under animal use protocol (AUP2363).

### Chemicals and reagents

Resveratrol (RES) was purchased from Supelco (MO, United States) and prepared freshly in an appropriate vehicle (VEH) consisting of 1% carboxyl methyl cellulose (CMC, Sigma-Aldrich, United States). SEB was obtained from Toxin Technology Inc. (FL, United States) and aliquoted in sterile phosphates buffer saline (PBS) at a stock concentration of 2 μg/μL prior to storage at −20°C until being used.

### Experimental design

We used a previously described method to induce ARDS by SEB involving double sensitization with SEB in C3H/HeJ mice which leads to 100% mortality and treatment with RES reverses this action. Briefly, C3H/HeJ mice pretreated orally with two doses of either 100 mg/kg resveratrol or VEH at 24 h intervals. After the second dose of RES, intranasal administration of 5µg/mouse of SEB was given and then after 90 min from the first SEB dose, a second dose of i. p 2µg/mouse of SEB was given as a fatal model of dual-dose SEB exposure (Rao et al., 2015b; Alghetaa et al., 2018). Endpoint of the experiment was 48 h post first SEB exposure at which time the mice were euthanized and the infiltrating mononuclear cells in the lungs were collected by gradient separation (Elliott et al., 2016).

### Mononuclear cell (MNC) isolation from the lungs and cell preparation from the spleens

Mononuclear cells (MNCs) from the lungs were prepared as described previously (Alghetaa et al., 2018; Mohammed et al., 2020b; Alghetaa et al., 2021; Sultan et al., 2021). Briefly, excised lungs were subjected to a mechanical tissue homogenizer (Seward, England). The tissue suspension was filtered and the single-cell mixture was suspended in cold fluorescence-activated cell sorting buffer (FACS) prepared from PBS enriched with 10% fetal bovine serum protein (FBS). RBC-lysis buffer (Sigma-Aldrich, United States) was used to remove the RBC from the mixture before being filtered with a 100 micron-strainer (Fisher Scientific, China). The filtered suspension was layered on Ficoll gradient, Histopaque-1077 (Sigma-Aldrich, United States) to separate MNCs. All MNC preparations were finally suspended in FACS and cell counts were measured by using trypan blue dye *via* auto cell counter 2000 (Bio-Rad, United States). Spleen cell preparations were carried out in a similar fashion except that they were not subjected to Ficoll gradient separation.

### Metabolome profiling and measurement of glycolysis and TCA metabolites using LC-MS

#### Sample preparation for mass spectrometry and metabolomics analysis

Metabolites were extracted from serum using the extraction procedure described previously (Wangler et al., 2017; Kornberg et al., 2018; Amara et al., 2019; Vantaku et al., 2019a; Vantaku et al., 2019b; Vantaku et al., 2019c). Briefly, 50 µl of serum sample was used for the metabolic extraction. The extraction step was started with the addition of 750 µL ice-cold methanol: water (4:1) containing 20 µL spiked internal standards to each cell pellet or tissue sample. Ice-cold chloroform and water were added at a 3:1 ratio for a final proportion of 1:4:3:1 water:methanol:chloroform:water. The organic (methanol and chloroform) and aqueous layers were mixed, dried, and resuspended with 50:50 methanol: water. The extract samples were deproteinized, followed by resuspension, and subjected to LC/MS analysis.

Ten µL of suspended samples were injected and analyzed using a 6,490 triple quadrupole mass spectrometer (Agilent Technologies, Santa Clara, CA) coupled to an HPLC system (Agilent Technologies, Santa Clara, CA) *via* single reaction monitoring (SRM).

#### Separation of glycolysis, TCA, and pentose pathway metabolites

Tricarboxylic acid cycle and Glycolysis cycle metabolites were identified by using 5 mM ammonium acetate in water as buffer PH 9.9 (A) and 100% acetonitrile as a buffer (B) using Luna 3 µM NH2 100 A^0^ Chromatography column (Phenomenex, Torrance, CA). The Gradient used: 0–20 min-80% B (Flow rate 0.2 ml/min); 20–20.10 min- 80% to 2% B; 20.10–25 min-2% B(Flow rate 0.3 ml/min); 25–30 min 80% B (Flowrate 0.35 ml/min); 30–35 min-80%B (Flow rate 0.4 ml/min); 35–38 min 80% B (Flow rate 0.4 ml/min); followed by re-equilibration at the end of the gradient to the initial starting condition 80% B a Flow rate of 0.2 ml/min. All the identified metabolites were normalized by spiked internal standard (Mohammed et al., 2020c).

### microRNA (miRNA) array and *in silico* analysis

miRNA analysis was carried out as described previously (Alharris et al., 2018; Neamah et al., 2019). Briefly, total RNA, including miRNA, was isolated from lung mononuclear cells using the miRNeasy kit from QIAGEN and following the protocol of the company. Microarray was performed using Affymetrix miRNA Array (version 4.1). Raw files generated from the miRNA microarray were uploaded to Gene Expression Omnibus (http://www.ncbi.nlm.nih.gov/geo) and deposited under accession number GSE220159. By using Transcriptome Analysis Console (TAC, ThermoFisher, United States), Log2 fold change of more than 3,000 miRNAs was detected from the raw array data, and only those miRNAs that were altered more than 2-fold were considered for further analysis. Filtered miRNAs were analyzed for their role in various biological pathways using Ingenuity Pathway Analysis (IPA) software http://www.ingenuity.com (Qiagen, Germany). Also, microRNA.org database was used to examine the sequence alignment regions between miR-100-5p and its targeted genes.

### Activation of spleen cells *in vitro* with SEB

Naïve spleens were excised from C3H/HeJ mice and processed to prepare a single cells suspension. The cells were seeded in U-bottom 96-well plates (Corning, United States) at a concentration of 5 × 10^5^ cells/well in complete DMEM medium (Sigma, United States) supplemented with 10% FBS (Atlanta for Biologicals, United States) 1% penicillin/streptomycin cocktail (Sigma, United States) and 50 µM mercaptoethanol (2-ME, Sigma, United States). Seeded cells were pretreated with either 50 µM RES or VEH for one hour before adding 1 μg/mL SEB to the culture. Activated cultures were incubated for 48 h at 37°C with 5% CO2 and the culture supernatants were collected for further analysis. Collected cultured cells were washed with phosphate buffer saline (PBS) twice before being suspended in FACS buffer. PE-conjugated anti-CD3 antibody (Biolegend, United States) was used to stain all harvested cells and then purified positively by using EasySep™ PE Positive Selection Kit II magnetic microbeads (STEMCELL, United States).

### ^3^H-thymidine incorporation assay

To evaluate the proliferative capacity of activated immune cells, SEB-activated MNCs from lungs or *in vitro* SEB-activated splenocytes were treated with thymidine (methyl-3H; PerkinElmer Health Sciences, United States) isotope at a concentration of 1µCi/well and incubated for 16 h to and then radioactivity was measured using a liquid-scintillation counter (MicroBeta TriLux; PerkinElmer, United States).

### RT-PCR assay

To determine the expression of Mtor, Pkm, Prkaa1, Rac1 and Rptor in the lung MNC or PE-selected T cells post-RES or vehicle treatment, RT-PCR was performed. The primer sequence of these genes was determined from the Primer Bank database and then built using Integrated DNA Technologies tools (Table 1). RT-PCR was performed for 40 cycles using the following conditions: 98°C (denaturing temperature) for 30 s, 98°C (annealing temperature) for 10 s, and 60°C (extension temperature) for 30 s. The expression of the above genes was normalized against mouse housekeeping gene Gapdh expression (internal control).

**TABLE 1:** Used primers with their Primer Bank identification number.

mRNA ID	Forward 5'----->3'	Reverse 5'----->3'	Primer bank ID
**Gapdh**	AGG​TCG​GTG​TGA​ACG​GAT​TTG	TGT​AGA​CCA​TGT​AGT​TGA​GGT​CA	6679937a1
**Mtor**	ACC​GGC​ACA​CAT​TTG​AAG​AAG	CTC​GTT​GAG​GAT​CAG​CAA​GG	9910228a1
**Pkm**	GCC​GCC​TGG​ACA​TTG​ACT​C	CCA​TGA​GAG​AAA​TTC​AGC​CGA​G	31981562a1
**Prkaa1**	GTC​AAA​GCC​GAC​CCA​ATG​ATA	CGT​ACA​CGC​AAA​TAA​TAG​GGG​TT	26349431a1
**Rac1**	GAG​ACG​GAG​CTG​TTG​GTA​AAA	ATA​GGC​CCA​GAT​TCA​CTG​GTT	13277918a1
**Rptor**	CAG​TCG​CCT​CTT​ATG​GGA​CTC	GGA​GCC​TTC​GAT​TTT​CTC​ACA	30061325a1

Gapdh, glyceraldehyde-3-phosphate dehydrogenase; Mtor, mechanistic target of rapamycin; Pkm, pyruvate kinase; Prkaa1, protein kinase, AMP-activated, alpha 1 catalytic subunit; Rac1, RAS-related C3 botulinum substrate 1 and Rptor, regulatory associated protein of MTOR.

### Transfection experiments with miR-100-5p mimic and inhibitor

Transfection of SEB-activated splenocyte cultures with miR-100-5p mimic and inhibitor was performed as previously described (Alharris et al., 2018). Briefly, C3H/HeJ naïve splenocytes were cultured in 24-well plates (2 × 10^5^ cells per well) and activated with 1 μg/mL of SEB for 24 h. Transfections were performed with Qiagen HiPerfect Transfection Reagent and 20 nmol/L of either syn-mmu-miR-100-5p mimic (Qiagen, MSY0000098), anti-mmu-miR-100-5p inhibitor (Qiagen, MIN0000098) or transfection reagent alone (mock, Qiagen). Transfection efficiency was validated with qRT-PCR for miR-100 (Qiagen, MS00031234), and expression of mTOR, Rac1, and Rptor after transfection was determined with RT-PCR (Mohammed et al., 2020a).

### Real-time PCR (RT-PCR) for detection of miR-100

Total RNA was isolated from lung mononuclear cells, cultured splenocytes, and miR-100-5p-transfected cells and then converted into cDNA by using miScript RT II kit (Qiagen, Germany) to prepare reverse transcription reaction component. After that, miR-100-5p-specific miScript Primer Assays (Qiagen, MS00031234, forward primer) were mixed with Universal Primer (reverse primer) and QuantiTect SYBR Green PCR Master Mix to quantify the enrichment of miR-100 in these prepared cDNAs. For accurate and reproducible results in miRNA quantification by real-time PCR, the amount of miR-100 was normalized by using an endogenous reference RNA consisting of Snord96A. The cycling conditions for RT-PCR were listed in Table 2.

**TABLE 2:** qRt-PCR cycle conditions for miRNA validation.

PCR cycle		Temp	Time	Repeat
Activation step		95C	15 min	N/A
3-step cycle	Denaturation	94C	15 s	40
	Annealing	55C	30 s	
	Extension	70C	30 s	

### Flow cytometry analysis of T cell subsets in the lungs

Single cell suspension of mononuclear cells was isolated from the lungs by using the gradient Ficoll layering method and prepared as described previously (Alghetaa et al., 2018). To avoid false positive or false negative immunofluorescence results, all cells were suspended in FACS buffer and incubated with FcγR blocker (Biolegend, United States). Then these cells were permeabilized (Pharmingen BD, United States) and stained with Biolegend anti-CD3, CD4, CD8, T-bet and IFN-γ antibodies conjugated with APC, BV786, PE, BV605 and APC-R700 fluorochromes respectively, which were used according to the manufacturer’s protocols.

### Immunofluorescence staining of mTOR

To validate the PCR results of upregulated mTOR gene expression, we performed immunofluorescent staining of lung tissue. We calculated the corrected total cell fluorescence (CTCF) for mTOR-stained cells in the lung tissue of naïve, SEB + VEH, and SEB + RES groups using ImageJ software from NIH, we performed immunofluorescent staining as described earlier (Alharris et al., 2018; Sarkar et al., 2019; Alharris et al., 2022). Briefly, the slides with a section of lung tissue were first deparaffinized according to the standard protocol, antigen retrieval was done using an antigen-retrieval solution from Abcam (Cambridge, MA, United States). The slides were then washed with PBS twice, permeabilized with 0.01% Triton X-100 (Sigma) for 15 min, washed three times with PBS for 5 min each, and then incubated overnight at 4°C with primary antibody (mouse-specific mTOR) from Abcam (Cambridge, MA, United States) diluted in 1% FBS in PBS. Next, the slides were washed with PBS three times for 10 min each and incubated with anti-mouse secondary antibody diluted in 1% FBS in PBS for 1 h at 37°C followed by washing the slides three times with PBS. The slides were then stained with DAPI to show the nuclei of the cells and washed three times with PBS and finally mounted with Antifade Mounting Medium from Vector Labs (Burlingame, CA). The tissue was visualized and imaged by usa ing Confocal microscope (Leica, United States).

### CellTrace assay of T cell division rate measured by carboxyfluorescein succinimidyl ester (CFSE) nuclear dye

Naïve splenocytes were prepared and suspended in 10 µM CSFE staining protein-free medium for 20 min, followed by staining the cells. The cells were washed twice before grouping them according to the treatment conditions and suspended in complete DMEM medium and seeded them at a concentration of 5 × 10^5^ cell/well in 96-well plates. Treated cells were then incubated for 48 h post 1 μg/mL SEB-activation at 37°C with 5% CO2 and then purified as described above to measure the cell division of T cells.

### Real-time metabolism analysis of T cells measured by seahorse XFp

Mitochondrial respiration, glycolytic rate, ATP rate, and fuel substrate dependency were measured based on the quantification of oxygen consumption rates (OCR), extracellular acidification rate, and proton efflux rate (PER). In all of these assessments, initially 2.5 × 10^5^ cells/well of purified T cells were seeded in XFp culture plates (Agilent, United States) by using CellTak cell tissue adhesive (Corning, United States). According to the assay recommendations, the cells were suspended in Agilent Seahorse XF DMEM Medium pH 7.4 (Agilent, United States) supplemented with 10 mM glucose, 1 mM sodium pyruvate, and/or 2 mM glutamine. Mitochondrial stress test, glycolysis stress test, ATP production rate test, glucose dependency test, and fatty acid dependency test were performed according to the manufacturer’s protocols. To achieve these measurements, a combination of 1 mM oligomycin, 1 mM fluorocarbonyl-cyanide-phenylhydrazone (FCCP), 0.5 mM rotenone, and antimycin-A and/or 2-deoxy-D-glucose (2-DG) were used according to the assay protocol. Once the assay was finished, all the cells in the run plate were stained with 2 µM final concentration of Hoechst 33,342 dye to quantify the live cell numbers by Cytation5 Imaging Cytometer (BioTek, United States) and to normalize the acquired data accordingly. All acquired data generated by Seahorse XFp were normalized and interpreted kinetically with Seahorse Wave Desktop Software (SWD) (Agilent, United States). Kinetic data of glycolytic and mitochondrial ATP production were generated by using Seahorse XF Real-Time ATP Rate Assay Kit according to the manufacturer’s protocol. Briefly, the alterations of concentrations of oxygen and proton in the culture medium due to the mitochondrial and glycolysis processes in living cells was detected *via* the chemical sensors. Oligomycin, an inhibitor of mitochondrial ATP synthase and rotenone/antimycin A chemicals were injected into the microplate to challenge the cellular respiration events by which the sensors calculate the delta of OCR and ECAR. All acquired data were then interpreted by using the SWD into bioenergetic kinetics. For detecting what type of fuel is utilized by mitochondria, Agilent Seahorse XF Mito Fuel Flex Test Kits was used. The assay medium consisting of DMEM for this test was supplemented with 1 mM pyruvate, 2 mM glutamate and 10 mM glucose and the medium pH was adjusted to be 7.4 at the time of analysis. The sensor cartridge was loaded with 30 µM bis-2-(5-phenylacetamido-1,2,4-thiadiazol-2-yl)ethyl sulfide (BPTES), an inhibitor of glutamine oxidation pathway, 40 µM Etomoxir, an inhibitor of long chain fatty acid oxidation, and 20µM UK5099, an inhibitor of glucose oxidation pathway. Afterwards, SWD was used to calculate the dependency, capacity and flexibility of T cells in switching among different sources of energy fuels.

### Single-cell RNA-seq (scRNA-seq) and analysis of lung cellular heterogeneity composition

The TC20 Automated Cell Counter (BioRad) was used to measure the cell count and viability of isolated lung cells. The cells were next loaded onto the Chromium Controller (10x Genomics). Following the manufacturer’s protocol, the Chromium single cell 5′ reagent kits (10x Genomics) were used to process samples into single-cell RNA-seq (scRNAseq) libraries. Sequencing of those libraries was performed using the NextSeq 550 instrument (Illumina) with a depth of 40k–60k reads per cell. The base call files generated from sequencing the libraries were then processed in the 10x Genomics Cell Ranger pipeline (version 2.0) to create FASTQ files. The FASTQ files were then aligned to the mm10 mouse genome and the read count for each gene in each cell was generated (Mohammed et al., 2020c). Downstream analysis was completed using Seurat suite version 3.0 within R studio (Butler et al., 2018; Stuart et al., 2019). Data were integrated into Seurat using anchor and integration functions. The integrated data were scaled and Principal-component analysis (PCA) was completed for dimensionality reduction. Clusters were made following PCA analysis by adjusting the granularity resolution to 0.25. We determined the number of principal components (PCs) to utilize post-JackStraw analysis within Seurat to determine PCs with the lowest *p*-value. Differential expressions were determined for each cluster to determine cluster biomarkers, and between the vehicle and resveratrol-treated samples using the default Wilcoxon rank sum test.

### Statistical analyses

In this study, we used groups of five mice treated with SEB + VEH or SEB + RES unless stated differently. The metabolome data were log2-transformed and normalized with internal standards on a per-sample, per-method basis. Statistical analyses were performed with a t-test in R Studio (R Studio Inc., Boston, MA). Differential metabolites were identified by adjusting the *p*-values for multiple testing at an FDR (Benjamini Hochberg method) threshold of <0.25. The ANOVA test was applied whenever there were more than two group comparisons with multiple Tukey’s corrections. The T-test was applied with the Holm-Sidak correction method to compare two groups. *p* < 0.05 was considered as significant threshold. Significant differences were depicted as *p < 0.05, **p < 0.01, ***p < 0.001, #*p* < 0.0001. Each experiment was performed three independent times unless stated differently.

## Results

### Resveratrol treatment in SEB-injected mice led to suppression of mTOR pathway via upregulation of miR-100

**FIGURE 1:**
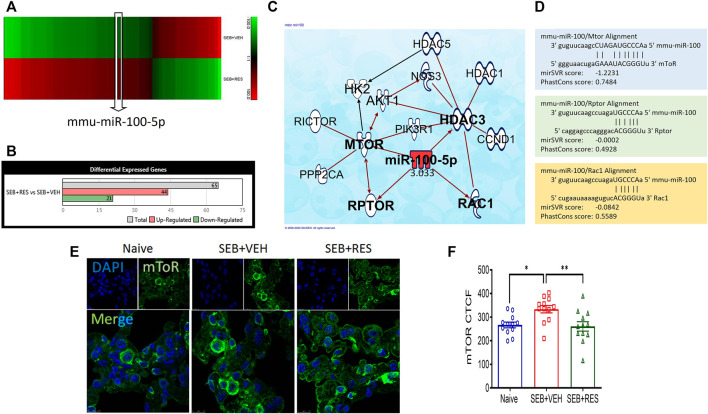
Effect of RES on miRNA induction and regulation of mTOR pathway in SEB-activated immune cells. Mice (n = 5) were exposed to SEB and treated with RES as described in Methods. Forty-eight hours later, mice were euthanized and studied as described below: **(A)**. Heat map showing altered expression of miRNAs in the lung mononuclear cells from SEB + VEH vs. SEB + RES groups. Five biological samples were pooled for the *in silico* analyses only, but all validations were performed on individual samples. **(B)**. Bar graph depicting counts of differentially expressed miRNAs with 2-fold change. **(C)**. Ingenuity pathway analysis (IPA) predicted the targeted genes by miR-100. **(D)**. Scheme of alignment regions between miR-100 and its targeted genes (mTOR, Rptor and Rac1). **(E)**. Immunofluorescent staining showing mTOR protein expression in the lung sections imaged by Leica confocal microscope and quantified by ImageJ software. **(F)**. Statistical analysis of mTOR protein expression. Compared values represent mean ± standard error of means (M±SE). One-way ANOVA test with multiple Tukey’s correction was applied. *p* < 0.05 considered as significant threshold. Significant differences were depicted as *p < 0.05, **p < 0.01.

Previous studies from our laboratory showed that RES is highly effective in preventing SEB-mediated ARDS (Alghetaa et al., 2018; Alghetaa et al., 2021). To investigate the potential role of miRNA in the amelioration effects of RES, we performed microRNA arrays. The majority of miRNAs (miR) in the lung’s MNCs showed altered expression when the mice were exposed to SEB and this effect was reversed significantly when RES was used (Figure 1A). When we considered those miRs with a >2-fold change (Figure 1A), we identified 21 miRNAs that were down-regulated and 44 miRs that were upregulated in the SEB + RES group in comparison to the SEB + VEH group (Figure 1B). By using IPA software to predict the target genes of these miRs, we were able to identify pathways that regulated cellular metabolism and proliferation and found that miR-100-5p was of interest because it was down-regulated by SEB exposure and reversed by RES treatment (Figure 1C). Interestingly, the pathway analysis suggested that miR-100-5p may target the mTOR signaling pathway as well as the Regulatory-associated protein of mTOR (RPTOR), and Rac family small GTPase 1 (Rac1). To further confirm the target genes, we used the bioinformatic tool, microrna.org, and found that miR-100-5p did target RPTOR, and Rac1 (Figure 1D). We also studied the mTOR protein expression in the lungs using the immunofluorescence technique and found that mTOR expression was significantly upregulated following SEB treatment when compared to the naïve group and furthermore, RES treatment caused a significant decrease in mTOR expression (Figures 1E,F).

### *In vivo* and *in vitro* treatment with RES leads to suppression of the mTOR gene via upregulation of miR-100-5p and to reduce energy production of SEB-activated T cells

**FIGURE 2:**
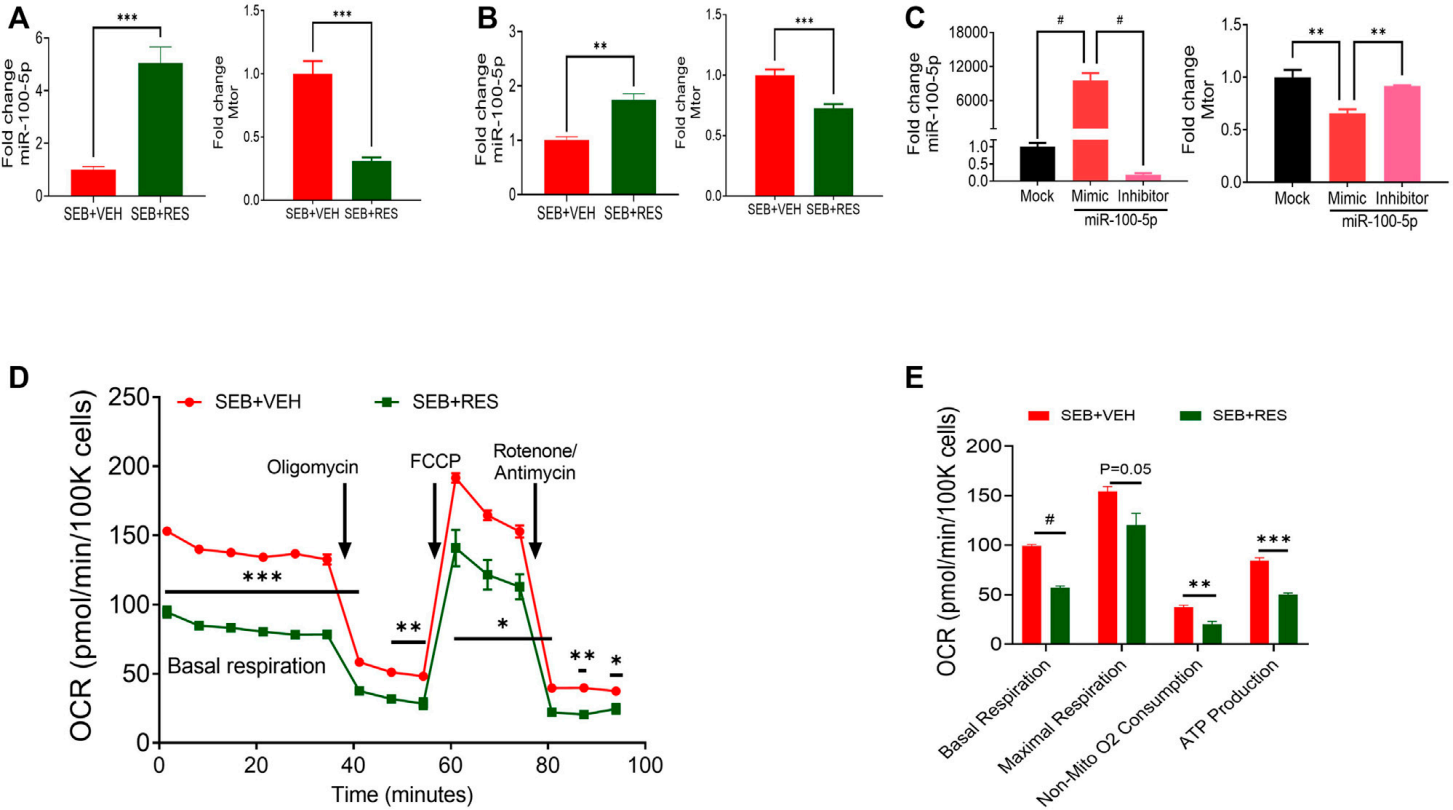
*In vivo* and *in vitro* treatment of RES leads to suppression of mTOR gene expression via upregulation of miR-100 and decreased energy production in SEB-activated T cells ***.*** Mice were treated with SEB and RES as described in Figure 1. **(A)** q-RT-PCR showing the levels of expression of miR-100 and mTOR genes in the lung MNCs. **(B)** q-RT-PCR analysis of miR-100 and mTOR gene expression in PE-selected CD3^+^ cells purified from splenocytes activated with SEB and treated with VEH or RES. **(C)** q-RT-PCR analysis of miR-100 and mTOR gene expression in transfected SEB-activated splenocytes with miR-100 mimic siRNA or inhibitor compared to mock-treated group. **(D)**. Real-time analysis of mitochondrial respiration in T cells by using Seahorse system in presence of oligomycin, Carbonyl cyanide-4-phenylhydrazone (FCCP) and combination of rotenone and antimycin. **(E)**. Quantification of selected CD3^+^ T cell respiratory kinetics calculated by report generator software based on changes in oxygen consumption rate (OCR) measured in panel **(D)**. Compared values represent mean ± standard error of means (M±SE). One-way ANOVA test with multiple Tukey’s correction was applied to compare more than two groups. T-test was applied with Holm-Sidak correction method to compare two groups. *p* < 0.05 considered as significant threshold. Significant differences were depicted as *p < 0.05, **p < 0.01, ***p < 0.001, #*p* < 0.0001.

Next, we directly examined the expression of mTOR and miR-100-5p in isolated lung MNCs and found that the SEB + RES group expressed higher levels of miR-100-5p and decreased levels of mTOR when compared to the SEB + VEH group (Figure 2A). Similar observations were made using SEB-activated *in vitro* cultures (Figure 2B). Transfection studies using mimic and inhibitor of miR-100-5p revealed the inverse relationship between the expression of miR-100-5p and mTOR gene expression (Figure 2C). The metabolic kinetics showed that the basal respiration rate, non-mitochondrial oxygen consumption rate, and ATP production rate were significantly higher in T cells of SEB + VEH than in the SEB + RES group (Figures 2D,E).

### Single-cell RNA-sequencing (scRNA-seq) reveals downregulation of glucose metabolism-related genes post-RES treatment

**FIGURE 3:**
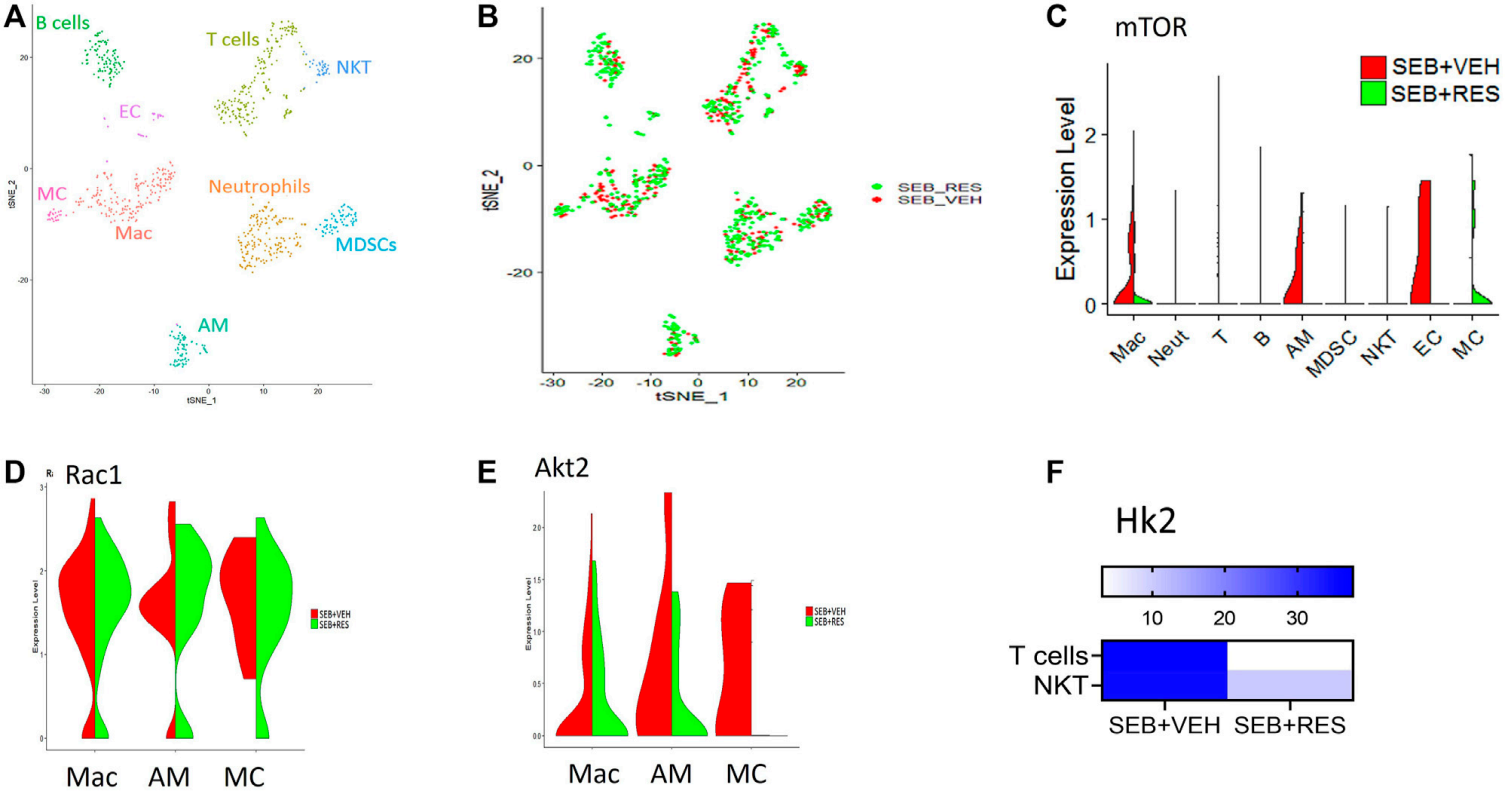
Single-cell RNA-sequencing (scRNA-seq) reveals downregulation of glucose metabolism-related genes post-RES treatment. Mice were treated as mentioned in Figure 1 legend. Whole lung tissues were excised and a single cell suspension was prepared. scRNA-seq was performed as described in Methods. The cells studied included: Mac: macrophages, Neut: Neutrophils, T: T cells, **(B)** B cells, AM: Alveolar macrophages, MDSC: Myeloid-derived suppressor cells, EC: Endothelial cells. MC: Monocytes. **(A)** scRNA-seq tSNE colored by cell phenotypes. **(B)** scRNA-seq tSNE colored by group ID consisting of SEB + VEH and SEB + RES. **(C)**. The violin plot depicts the Mtor gene expression levels and enrichments in different lung cellular components. **(D)**. Violin plot depicts Rac1 gene expression levels and enrichment. **(E)**. The violin plot depicts Akt2 gene expression levels and enrichment. **(F)**. Heatmap expression of Hk2 in T cells and NKT cells.

scRNA-seq was used to unveil the lung cellular components. We found that the majority of resident and infiltrating immune cells were natural killer T cells (NKT), macrophages (Mac), alveolar macrophages (AM), and myeloid-derived suppressor cells (MDSCs) where each cell phenotype was clustered based on their similarity of transcriptomic profile (Figure 3A). Also, the T-distributed Stochastic Neighbor Embedding (tSNE) plot was used to visualize the different cell phenotypes in each study group colored by group ID (Figure 3B). The violin plots showed that the mTOR was highly expressed in macrophages (Mac), alveolar macrophages (AM), and endothelial cells (EC) in the SEB + VEH group and it was significantly decreased in the SEB + RES group (Figure 3C). Furthermore, violin plots also showed other genes related to energy metabolism such as Rac1 (Figure 3D) and Akt2 (Figure 3E), in which Rac1 was increased while Akt2 was decreased in some of the cells examined from the SEB + RES group when compared to the controls. There was also a significant reduction in hexokinase two enzyme (Hk2), a key enzyme of glucose metabolism, in T cells in general and NKT populations of the SEB + RES group in comparison with the SEB + VEH group (Figure 3F).

**FIGURE 4:**
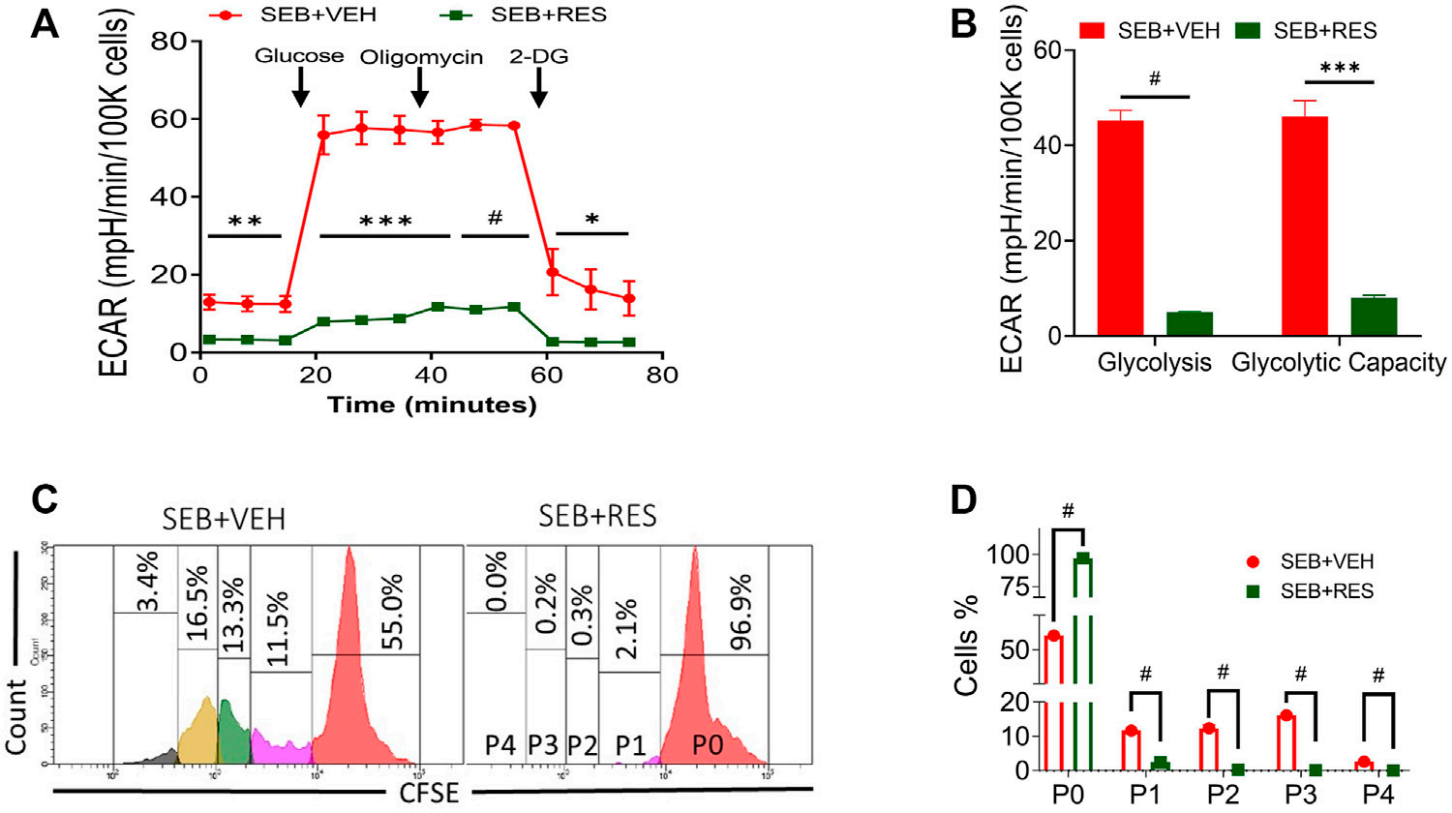
Effect of RES on glycolysis and proliferative capacity of SEB-activated T cells. Mice were treated as mentioned in Figure 1 legend. Whole lung tissues were excised and the single cell suspension was prepared for real-time metabolism analysis. **(A)**. Real-time analysis of T cell glycolysis measured by Seahorse system based on the calculation of extracellular acidification rate (ECAR) changes. **(B)**. Glycolysis statistical kinetics of T cells calculated by using Report Generator software. **(C)**. CFSE staining follows T-cell division *in vitro*. Naïve splenocytes were incubated with CFSE dye at a concentration of 10 μM for 20 min at room temperature before being washed and treated with RES or VEH and then activated with 1 μg/ml SEB for 48 h. P, parent cells, 0–4 sequence of cell division. **(D)**. Statistical analysis of T cell divisions depicted as P0-P4. Compared values represent mean ± standard error of means (M ± SE). One-way ANOVA test with multiple Tukey’s correction was applied to compare more than two groups. T-test was applied with Holm-Sidak correction method to compare two groups. *p* < 0.05 considered as significant threshold. Significant differences were depicted as *p < 0.05, **p < 0.01, ***p < 0.001, #*p* < 0.0001.

The findings that Hk2 is inhibited led us to examine the glycolysis rate of glucose in SEB-activated T cells treated with RES or VEH by using the Seahorse system, a real-time metabolism analyzer. Interestingly, we found that RES-treated T cells significantly lowered their glycolysis capacity measured by extracellular acidification rate (ECAR) before and after being challenged with glucose (Figure 4A). According to the kinetic calculation of the metabolic behavior of study groups, the glycolysis rate and glycolytic capacity of SEB-activated T cells treated with VEH were significantly higher than in of SEB + RES group (Figure 4B). All the above changes drove us to examine the cell division of SEB-activated T cells in presence of RES or VEH by using CSFE, a permeable dye. We found that SEB-activated T cells were able to continue in the biological cell divisions and divided for up to four new generations within 48 h into SEB + VEH treated cells (Figures 4C,D), while SEB + RES treatment blocked the cell division (Figures 4C,D).

### Effect of RES treatment on reprogramming of SEB-activated T cell mitochondrial function from fatty acid biosynthesis into fatty acid β-oxidation

**FIGURE 5:**
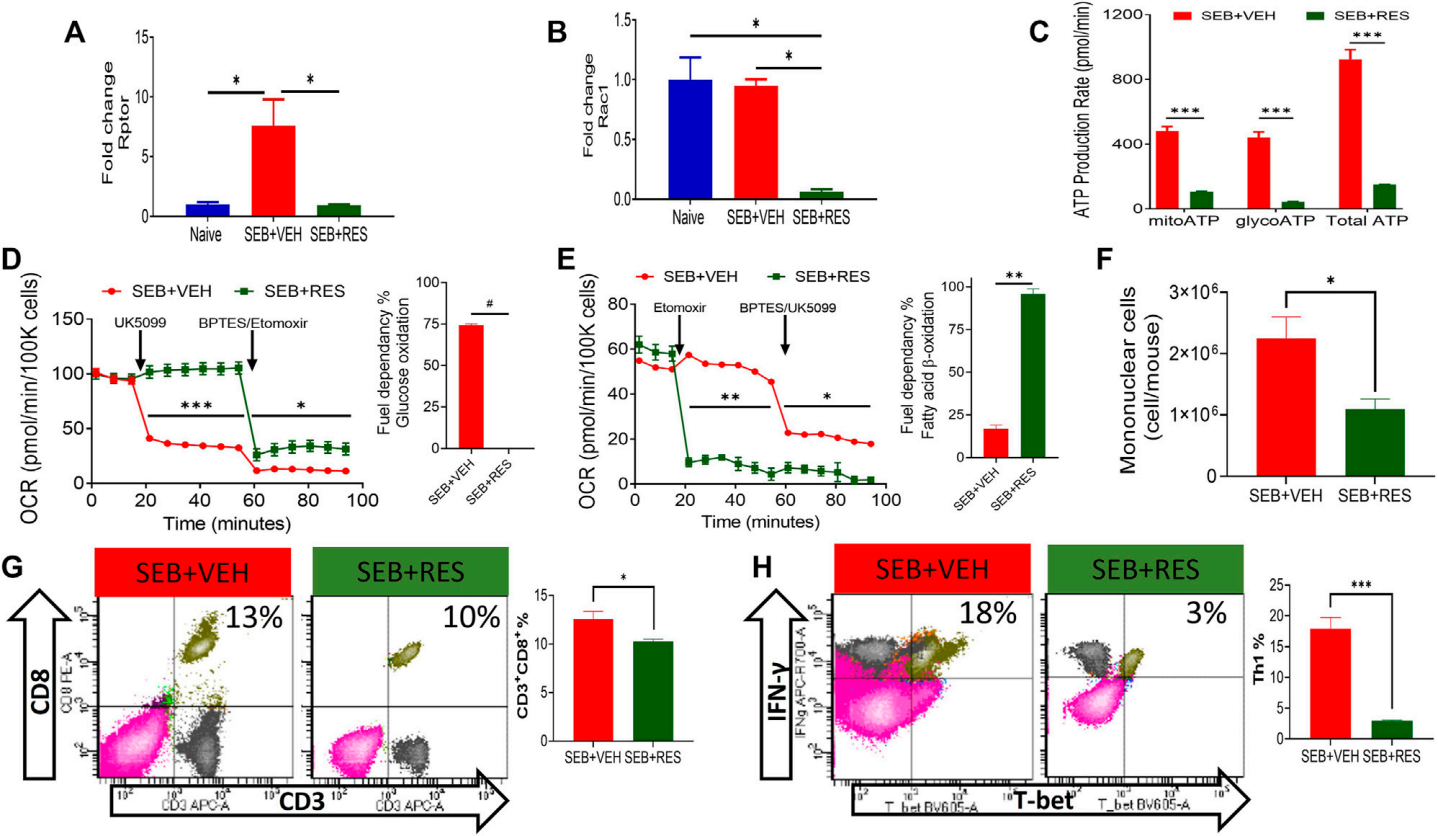
Upregulation of miR-100 by RES treatment leads to reprogramming of SEB-activated T cell mitochondrial function from fatty acid biosynthesis into fatty acid β-oxidation. Mice were treated with SEB + VEH or SEB + RES as mentioned in Figure 1 legend. Total RNA isolated from lung MNC was further analyzed. **(A,B)**. Real-time qPCR quantification of Rptor and Rac1 gene expression in the MNCs isolated from the lungs. **(C)**. Real-time kinetic data in live cells showing ATP production by mitochondria (mitoATP), glycolysis (glycoATP) and total ATP in purified T cells. **(D,E)**. Mitochondrial fuel substrate dependency tests detected by Seahorse real-time metabolism analyzer. Glucose **(D)** and Fatty acid **(E)** cellular metabolism in purified T cells. **(F)** Mononuclear cells count per mouse (lung set) isolated from lungs by gradient separation. **(G)** Flow cytometric analysis of CD3^+^CD8^+^ cells gated on singlet population in lung mononuclear cells with a bar graph of statistical analysis. **(H)** Flow cytometry of T-bet^+^IFN-γ^+^ cells gated on CD3^+^CD4^+^ cells in lung mononuclear cells with a bar graph of statistical analysis. Compared values represent mean ± standard error of means (M±SE). One-way ANOVA test with multiple Tukey’s correction was applied to compare more than two groups. T-test was applied with Holm-Sidak correction method to compare two groups. *p* < 0.05 considered as significant threshold. Significant differences were depicted as *p < 0.05, **p < 0.01, ***p < 0.001, #*p* < 0.0001.

We also tested the levels of mTOR signaling pathway molecules and found that Rptor and Rac1 were found to be significantly suppressed in lung MNCs in SEB + RES-treated mice when compared to the SEB + VEH group (Figures 5A,B). Because mTOR is the major regulator of energy production in mitochondria and cell proliferation (Morita et al., 2015), we also studied this effect. We noted that there was a significant reduction in the total production of ATP molecules either *via* glycolysis or mitochondrial oxidation (Figure 5C). We found that inhibiting glucose mitochondrial oxidation by using UK5099, a selective mitochondrial pyruvate carrier (MPC) inhibitor, did not change the mitochondrial glucose oxidation rate in T cells from the SEB + RES group, while it caused a significant drop in the mitochondrial oxidative respiration in SEB + VEH, which suggested that these cells were glucose-dependent on their energy production in SEB-activation model (Figure 5D). Furthermore, efforts to detect the alternative fuel substrate utilized by RES-treated SEB-activated T cells led to the finding that fatty acid β-oxidation may be the main pathway of mitochondrial energy production in this group when compared to the SEB + VEH group (Figure 5E). Finally, we found that metabolic reprogramming of infiltrating and resident mononuclear immune cells by RES treatment was associated with a significant reduction in the number of these cells in the lungs of SEB-injected mice (Figure 5F). This reduction in the number of mononuclear cells was accompanied also by a statistical reduction in the effector T cell populations in the lungs of SEB-injected mice treated with RES such as the CD3^+^CD8^+^ (Figure 5G) and inflammatory IFN-γ^+^Th1^+^ cells (Figure 5H).

### Resveratrol-mediated cell-metabolism reprogramming is associated with a reduction in the proliferative activity of SEB-activated immune cells

**FIGURE 6:**
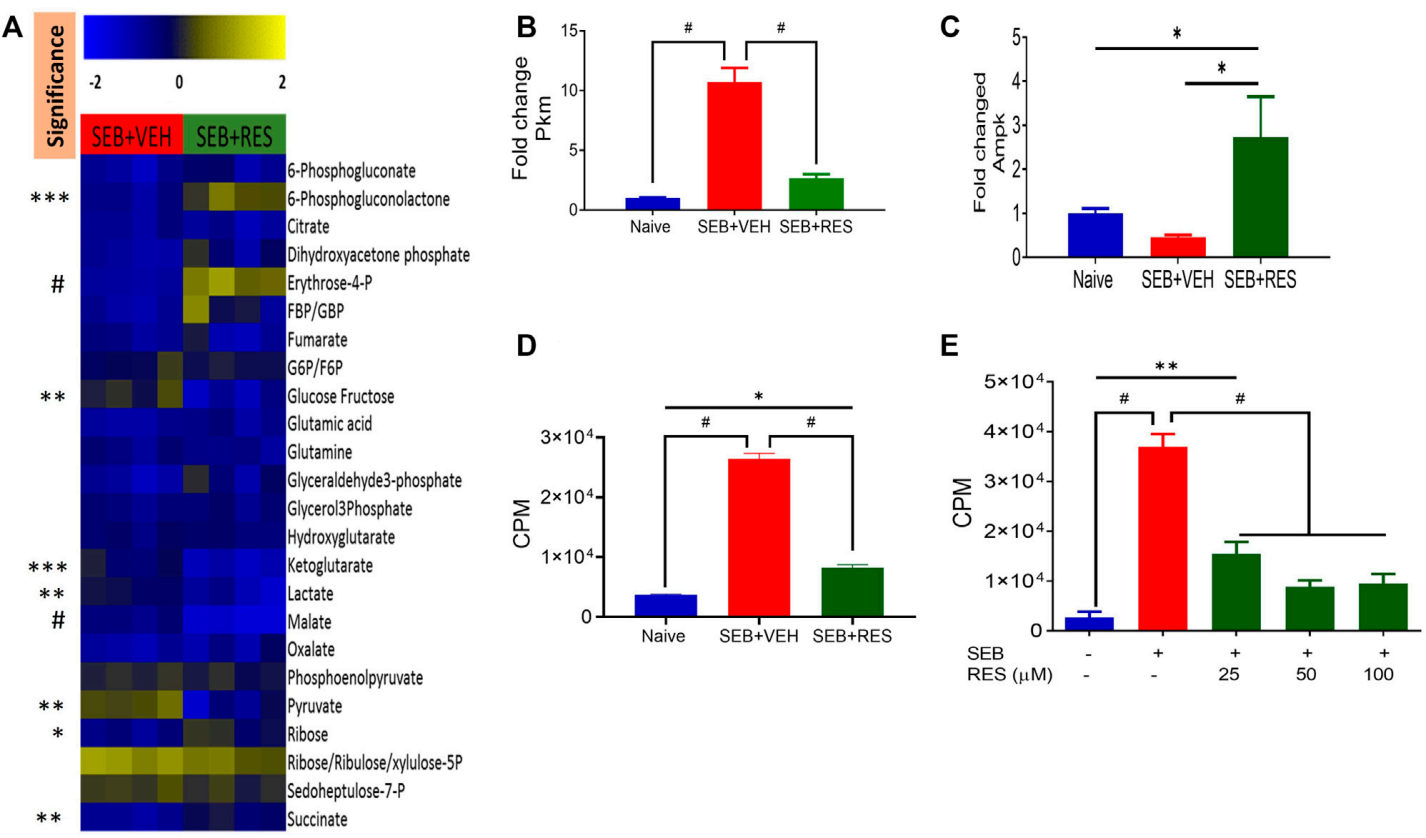
Resveratrol-mediated cell metabolism reprogramming leads to a reduction in the proliferative capacity of SEB-activated immune cells**.** Mice were treated with SEB + VEH or SEB + RES as described in Figure 1. **(A)**. LC-MS results showing serum metabolomic profile (n = 4). **(B)**. Real-time qPCR results of Pkm gene expression in the lung MNC. **(C)**. Real-time qPCR of Ampk gene expression in the lung MNC. **(D)**. Lung mononuclear cells were isolated by gradient separation from different study groups and then seeded in a 96-well plate and incubated with one μCui/well ^3^H-thymidine for 16 h. CPM: Counts per minute. **(E)**. Naïve splenocytes pretreated with RES one hour prior to 1 μg/mL SEB activation for 48 h, and incubated with ^3^H-thymidine for 16 h to determine isotope incorporation. Compared values represent mean ± standard error of means (M±SE). One-way ANOVA test with multiple Tukey’s correction was applied. *p* < 0.05 considered as significant threshold. Significant differences were depicted as *p < 0.05, **p < 0.01, ***p < 0.001, #*p* < 0.0001.

To further characterize the metabolic pathways, we used LC-MS to determine glucose-derived metabolites in the serum. Interestingly, we found significant upregulation of glucose, α-ketoglutarate, lactate, malate, and pyruvate in the sera of the SEB + VEH group when compared to the SEB + RES group (Figure 6A). The elevated levels of glucose metabolism intermediates were associated with a significant increase in the gene expression of pyruvate kinase enzyme (Pkm) in the lung’s infiltrating mononuclear cells (MNC) when compared to naïve or SEB + RES groups (Figure 6B). To detect if this downregulation of glucose metabolism by RES could have an impact on the adenosine triphosphate production, we looked at the expression of AMP-activated protein kinase (Ampk), the gene that regulates cellular energy homeostasis, largely to activate glucose and fatty acid uptake and oxidation when cellular energy is low. We found that the gene expression of Ampk was significantly higher in the MNC of the SEB + RES group than in naïve or SEB + VEH groups (Figure 6C). It is known that when Ampk is highly expressed this will arrest most of the cellular biological functions in these affected cells. We found that RES treatment *in vitro* caused a significant reduction in the proliferative capacity of SEB-activated MNCs when compared to the SEB + VEH group (Figures 6D,E).

## Discussion

Immunometabolism is one of the most exciting areas of translational research that has been studied extensively during the past decade. It refers to the metabolic processes that regulate different immune cell responses in a healthy state as well as during inflammatory responses. Manipulating immunometabolism could pave the way for novel therapies for acute and chronic inflammation as well as autoimmune diseases *via* anabolic and catabolic processes to build or use adenosine tri-phosphate (ATP), respectively (O'Neill et al., 2016).

In the current study, we treated the mice with RES before SEB administration suggesting that RES may be useful as a preventive strategy. This was necessary because SEB being a superantigen, activates a significant proportion of T cells thereby causing a massive cytokine storm within a short period (Mohammed et al., 2020a). Our studies are relevant because SEB is considered a biological warfare agent (Madsen, 2001) and thus, preventive strategies are necessary at the population level. Moreover, with milder forms of ARDS, RES may still be effective when given after the onset lung inflammation. For example, in our previous studies using Ova-induced asthma and lung inflammation, RES was effective when given after the administration of the antigen (Alharris et al., 2022). It is noteworthy that RES is readily available as a dietary supplement in the market and it remains to be established if people who use it are less likely to develop lung inflammation following exposure to external agents.

In the current study, we found that miR-100-5p was upregulated in MNCs isolated from the lungs of mice treated with SEB + RES. Additionally, we found that miR-100-5p regulated the expression of the mTOR signaling pathway. mTOR plays a central role in gene transcription, cell apoptosis, proliferation and differentiation, angiogenesis, tumor formation and development, and cell senescence through PI3K/Akt/mTOR signaling pathway (Hassan et al., 2013), and inhibiting this pathway could have therapeutic promises in leukemia disorders (Bertacchini et al., 2015). The precise role of miR-100-5p in the regulation of inflammation is not clear. Some recent studies suggested that miR-100-5p may play a role in neurodegeneration (Wallach et al., 2021). It was shown that miR-100-5p released from apoptotic cortical neurons, may serve as an endogenous TLR7/8 ligand, leading to neuronal apoptosis (Wallach et al., 2021). The miR-100-5p expression has also been studied in cancer models. In one study, it was shown that miR-100-5p was expressed at lower levels in prostate cancer cells which was associated with increased expression of mTOR thereby affecting the proliferation, migration, and invasion of tumor cells (Ye et al., 2020).

The mTOR has been shown to target a serine/threonine kinase that regulates growth, proliferation, survival, and autophagy based on the nature of the cells involved (Perl, 2016). mTOR forms two interacting complexes, mTORC1, and mTORC2. The latter has been shown to promote the expansion of proinflammatory Th1 and Th17 cells thereby promoting the pathogenesis of autoimmune diseases (Perl, 2016). Thus, the blockade of mTOR signaling is considered to be a potential therapeutic approach to treating inflammatory diseases. It is thus interesting to note that RES which is well characterized for its anti-inflammatory properties may mediate these effects through suppression of the mTOR signaling pathway.

Upon SEB induction of ARDS, we found significantly increased glycolysis and tri-carboxylic acid (TCA) metabolites in the sera of SEB + VEH mice accompanied by an increase in PKM gene in the lung MNCs while suppressing AMPK gene expression. Interestingly, treatment with RES reversed the expression patterns of these genes. Pyruvate kinase (PK) is a key enzyme that catalyzes the dephosphorylation of phosphoenolpyruvate into pyruvate and regulates the production of ATP during glycolysis (Liu et al., 2022). Pyruvate kinase M2 (PKM2) is an important isozyme of PK, which is expressed in immune cells and has been shown to increase the expression of the proinflammatory cytokines (Liu et al., 2022). Thus, RES by decreasing the expression of PK may help suppress inflammatory cytokines induced by SEB. Additionally, PKM2 can also activate mTORC1 by phosphorylating mTORC1 inhibitor AKT1 substrate 1 (AKT1S1) (He et al., 2016).

AMPK and mTOR are two critical kinases that together regulate every aspect of cellular and systemic metabolism, they act as sensors to harmonize the cellular energy and available nutrient levels (Sukumaran et al., 2020). We noted that RES caused an increase in AMPK expression. It is interesting to note that AMPK is also shown to mediate anti-inflammatory effects through activation of SIRT1, PGC-1α, p53, FoxO3a, and p300, and down-regulating the activity of various inflammatory molecules such as NF-κB and AP-1 (Liu et al., 2018). Thus, RES-mediated increase in AMPK may also directly suppress SEB-mediated inflammation through pathways indicated above. Activated AMPK has also been shown to inhibit mTORC1 signaling (Agarwal et al., 2015) as a result of glucose deprivation which leads to a significant reduction in mitochondrial biogenesis (O'Neill et al., 2016; Poznanski et al., 2018; Leprivier and Rotblat, 2020). Because AMPK is activated by metabolic stress and many drugs and xenobiotics, this leads to switching off the anabolic functions of the cells that require ATP consumption and thereby affecting the cell cycle progress (Hardie et al., 2012; Rabinovitch et al., 2017) and limits the ability of the cells to synthesize the required precursors for cell division, consistent with our findings that RES-exposed cells exhibited less proliferation when activated by SEB. Furthermore, RES treatment of SEB-injected mice led to downregulation of the Rac1 gene, a member of the Rho family of GTPases, in the MNCs isolated from the lungs. RAC1 has been shown to regulate cellular glucose uptake through cell membrane glucose transporters (Sylow et al., 2016). Thus, the decrease in Rac1 expression by RES was also associated with the inhibition of HK2, a glycolysis key enzyme, in the lung infiltrating T cells and NKT cells.

One of the limitations of this study was that we used only female mice. It is well-known that the estrogen receptor is a major modulator of immune response and the superantigen can initiate hyperactive effector cells significantly to a greater level in females versus males (Faulkner et al., 2007). Also, RES is known to act as a phytoestrogen and bind to estrogen receptors (Gehm et al., 1997). Moreover, it acts as an antagonist for the Aryl hydrocarbon receptor (Casper et al., 1999)). Both these receptors and their agonists are known to behave differentially in male vs. female mice (Zuloaga et al., 2014; Lee et al., 2015). It is for these reasons, we included only the female mice, and not a mixture of males and females in the current study, although similar studies need to be undertaken in the male mice as well.

**FIGURE 7:**
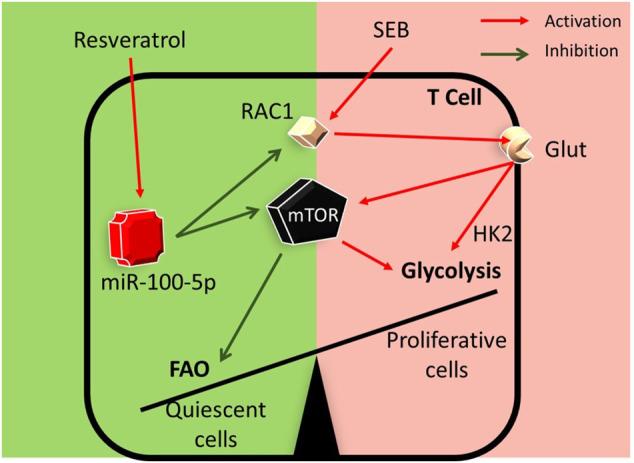
Graphical abstract: RES may suppress SEB-mediated hyperimmune response through the following pathway: RES induces miR-100 in activated immune cells which leads to decreased expression of Mtor, Rptor, and Rac1 genes. During T-cell activation, Mtor induces the transcription of many key glycolytic enzymes. mTOR regulates metabolic pathways through the activation of downstream transcriptional factors. Such transcription factors play a key role in the metabolic switch by promoting the expression of enzymes involved in aerobic glycolysis and other anabolic pathways involved in T cell activation and differentiation (Palmer et al., 2015). Thus, RES by suppressing mTOR pathway may alter the metabolic programming in SEB-activated T cells.

In summary, in the current study, we demonstrate a key mechanism through which RES may suppress SEB-mediated hyperimmune response in the lungs. This pathway involves the induction of miR-100-5p which in turn targets mTOR and decreases its expression. Additionally, while miR-100-5p may also target Rptor and Rac1, key molecules involved in mTOR signaling, based on *in silico* analysis, further studies are necessary to corroborate this. mTOR plays a key role in T cell activation and differentiation through nutrient sensing and antigen-receptor signaling (Salmond, 2018). Thus, RES by suppressing the mTOR pathway may alter the metabolic programming in SEB-activated T cells thereby suppressing the T cells from dividing and returning to a more quiescent stage (Figure 7).

## Data Availability

The datasets presented in this study can be found in online repositories. The names of the repository/repositories and accession number(s) can be found below: https://www.ncbi.nlm.nih.gov/geo/, GSE220159.

## Abbreviations

ARDS, acute respiratory distress syndrome; ECAR, extracellular acidic rate; OCR, oxygen consumption rate; PER, proton efflux rate; RES, resveratrol; SEB, *staphylococcus* enterotoxin-B; VEH, vehicle.
